# Adults with Greater Weight Satisfaction Report More Positive Health Behaviors and Have Better Health Status Regardless of BMI

**DOI:** 10.1155/2013/291371

**Published:** 2013-06-03

**Authors:** Christine E. Blake, James R. Hébert, Duck-chul Lee, Swann A. Adams, Susan E. Steck, Xuemei Sui, Jennifer L. Kuk, Meghan Baruth, Steven N. Blair

**Affiliations:** ^1^Department of Health Promotion, Education, and Behavior, Arnold School of Public Health, University of South Carolina, Columbia, SC 29208, USA; ^2^South Carolina Statewide Cancer Prevention and Control Program, University of South Carolina, Columbia, SC 29208, USA; ^3^Department of Epidemiology and Biostatistics, Arnold School of Public Health, University of South Carolina, Columbia, SC 29208, USA; ^4^Department of Exercise Science, Arnold School of Public Health, University of South Carolina, Columbia, SC 29208, USA; ^5^School of Kinesiology and Health Science, York University, Toronto, ON, Canada M3J 1P3

## Abstract

*Background.* Prior studies suggest that weight satisfaction may preclude changes in behavior that lead to healthier weight among individuals who are overweight or obese. *Objective.* To gain a better understanding of complex relationships between weight satisfaction, weight-related health behaviors, and health outcomes. *Design.* Cross-sectional analysis of data from the Aerobics Center Longitudinal Study (ACLS). *Participants.* Large mixed-gender cohort of primarily white, middle-to-upper socioeconomic status (SES) adults with baseline examination between 1987 and 2002 (*n* = 19,003). *Main Outcome Variables.* Weight satisfaction, weight-related health behaviors, chronic health conditions, and clinical health indicators. *Statistical Analyses Performed.* Chi-square test, *t*-tests, and linear and multivariate logistic regression. *Results.* Compared to men, women were more likely to be dieting (32% women; 18% men) and had higher weight dissatisfaction. Men and women with greater weight dissatisfaction reported more dieting, yo-yo dieting, and snacking and consuming fewer meals, being less active, and having to eat either more or less than desired to maintain weight regardless of weight status. Those who were overweight or obese and dissatisfied with their weight had the poorest health. *Conclusion.* Greater satisfaction with one's weight was associated with positive health behaviors and health outcomes in both men and women and across weight status groups.

## 1. Introduction

Weight satisfaction has been shown to be associated with self-reported healthy lifestyle behaviors and with less intention to change future physical activity, diet, or body weight, even among overweight and obese adults [1]. Body mass index ((BMI) = weight(kg)/height(m)^2^), a proxy for adiposity, is related to a variety of health outcomes ranging from cause-specific and all-cause mortality, [2–4] to the incidence of numerous chronic diseases, including cancers of various sites [2–8]. 

Among individuals who are overweight or obese the combination of positive perceptions of weight status and negative intentions toward behavior change may preclude changes in behavior that lead to healthier weight and better weight-related health outcomes. Furthermore, it has been suggested that weight dissatisfaction may be beneficial in prompting individuals to improve health practices [9, 10]. Weight dissatisfaction, however, is an important “driver” of unhealthy dieting behaviors. Yo-yo dieting and its correlate, weight cycling, also are common in the population and are associated with a variety of health-related endpoints, including incident diabetes, [11] hypertension, [12] insulin sensitivity, [13] and mortality [14, 15]. One of the major determinants of dieting is weight dissatisfaction; however, there are few data on the association between weight satisfaction and health outcomes, particularly in populations including those of normal weight [1, 16]. A better understanding of relationships between weight satisfaction or dissatisfaction and weight-related health behaviors and health outcomes could provide important insights into these complex relationships. Using a large cohort in which data on many health-related variables were collected, we sought to examine relationships between weight satisfaction and a number of weight-related health behaviors, particularly dieting and the tendency to eat past satiety, chronic health conditions, and clinical health indicators among normal weight, overweight, and obese men and women.

## 2. Methods

### 2.1. Study Population

We analyzed data from the Aerobics Center Longitudinal Study (ACLS), a large cohort study of healthy adults. This study was reviewed and approved by the Cooper Institute Institutional Review Board on an annual basis. The sample for the current analysis consisted of 19,003 (14,408 men and 4,595 women) primarily white (>97%), well-educated (>75% college graduates), and middle-to-upper SES adults (most in executive and professional occupations). This cross-sectional study used data from participants' first clinical examination only in order to reduce possible confounding related to the reasons of/motivation for subsequent visits.

To be eligible for these analyses, subjects of either gender needed to be between the ages of 20 and 83 years, have undergone a clinical examination between 1987 and 2002, and have complete data on systolic and diastolic blood pressure, total cholesterol, fasting glucose, objectively measured weight, goal weight, eating habits, and dieting frequency. Subjects included in the analyses had no prior history of ulcer, gallbladder disease, jaundice, hepatitis, cirrhosis, or colon polyps. Additionally, all participants completed a treadmill exercise test to at least 85% of their age-predicted maximal heart rate (220- (age in years) beats per minute) [17]. As a further quality control measure, subjects whose height was outside of the range of 120 to 209 cm or whose body mass index (BMI = weight(kg)/height(m)^2^) was <18.5 or ≥50 kg/m^2^ were excluded (*n* = 228).

### 2.2. Clinical Examination

Participants who provided written informed consent to participate in the study arrived for the clinical examination after an overnight fast of at least 12 hours. Information was collected on personal and family health histories, fasting blood chemistry, anthropometry, resting blood pressure and electrocardiogram, and a maximal graded exercise test. Examination methods and procedures followed a standard manual of operations, as described previously [18]. BMI was computed from measured weight and height. Goal weight was assessed based on a question at the clinical examination: “What do you consider a good weight for yourself?” Goal relative weight (or goal BMI) was calculated from goal weight and measured height (goal weight(kg)/height(m)^2^). Weight satisfaction was defined as measured weight minus the goal weight, and relative weight satisfaction was computed as measured BMI minus the goal BMI. Eating to maintain weight was classified as less (much less or somewhat less), just right, or more (somewhat more or much more) based on a question: “some people have to watch what they eat all the time to control their weight, others eat all they want and their weight is fine, and others have to eat more than they want to keep their weight up. What is your case?” Dieting frequency was classified as less (never, rarely, or sometimes) or more (often or always) based on a question: “How often are you dieting (eating less than you would like)?” Yo-yo dieting was classified as “yes” or “no” based on the questions: “Are you a yo-yo dieter (do you intentionally lose weight, and then regain the weight often)?” Yo-yo dieting information was not available from the whole cohort and was not measured at baseline; it was added into the analytic dataset from a subgroup of 1565 participants who completed a mail-back survey in 1990. 

Resting blood pressure was recorded as the first and fifth Korotkoff sounds by auscultatory methods. Serum samples were analyzed for lipids and glucose using standardized automated bioassays by a laboratory that participates in the CDC Lipid Standardization Program and meets its quality control standards. Information on smoking habits (current, former, or nonsmoker), alcohol intake (grams per day), personal history of myocardial infarction, stroke, cancer, hypertension, diabetes, hypercholesterolemia, ulcer, gallbladder disease, jaundice, hepatitis, cirrhosis, colon polyps, goal weight, eating habits, and dieting frequency was obtained from a standardized medical history questionnaire administered at study entry. Self-assessment of physical activity was ascertained in the baseline survey and consisted of questions on current moderate and vigorous physical activity and intention regarding future activity [18].

 Cardiorespiratory fitness (CRF) was assessed with a maximal treadmill exercise test using a modified Balke protocol [18, 19]. Maximal metabolic equivalents (METs, 1 MET = 3.5 ml O_2_ uptake · kg^−1^· min^−1^) were estimated from the final treadmill speed and grade [20].

## 3. Statistical Methods 

All analyses were performed for men and women separately. Frequencies, means, and standard deviations were calculated for key variables, including selected demographic and health-related variables. Chi-square test for categorical variables and *t*-test for continuous variables examined differences between men and women on key variables. Based on the median value of the difference between actual and goal weight in men (4.08 kg) and women (3.97 kg) we dichotomized weight satisfaction into either satisfied (lower values) or dissatisfied (higher values). The relationships between weight satisfaction and a number of health behaviors (e.g., dieting, tendency to eat past satiety, eating to maintain weight, and measured body weight, and frequency of dieting), chronic health conditions (e.g., hypertension, diabetes), and health indicators (e.g., fitness, body fat) were examined first using chi-square test for categorical variables and *t*-test for continuous variables. Next, to examine whether health behaviors, chronic conditions, and health indicators differed across BMI categories within each weight satisfaction group (i.e., dissatisfied and satisfied) and within BMI categories across weight satisfaction groups we used chi-square test for categorical variables, and *t*-test or linear regression for continuous variables. 

All results for statistical tests were obtained using SAS software (version 9.2). *P* values presented are 2-sided. Rather than set an arbitrary level at which to judge a result as “significant,” exact *P* values are presented. These are rounded to the first nonzero numeral to the right of the decimal point for any 0.01 ≤ *P *> 0.0001. For *P *> 0.01, values are provided to two digits to the right of the decimal point. 

## 4. Results

Differences between men and women on all demographic and health-related variables evaluated using chi-square and *t*-tests were significant (Table 1). In particular, compared to women, men tended to be more overweight (mean BMI 23.3 versus 26.7 kg/m^2^), had higher rates of hypertension (16% versus 33%) and hypercholesterolemia (24% versus 31%), had higher rates of tobacco use (7% versus 14%), were more likely to report having to eat more to maintain their weight (10% versus 13%), were less likely to report dieting (32% versus 18%) more likely to eat fewer than 3 meals per day (60% versus 67%), and were less likely to be dissatisfied with their weight.

As is evident in Table 1, both men and women in the ACLS cohort have a goal weight much lower, on average (i.e., 5.8 and 6.2 kg, respectively, in men and women), than their measured weight. As a proportion of actual weight, women's goal weight was 10% lower than their measured weight, while men's goal weight was nearly 7% lower. In examining the relationships between weight satisfaction and weight-related health behaviors and weight-status categories we found that men and women who were dissatisfied with their weight were more likely to be overweight and obese (*P* < .0001). Despite much higher relative weights in men than women in this cohort, when examining differences in weight status relative to satisfaction (results not tabulated) we observed that a much higher proportion of normal-weight women (36%) than men (10%) were dissatisfied with their weight. Very few overweight or obese women (3%) were satisfied with their weight, whereas 27% of overweight or obese men were satisfied with their weight (*P* for all of these contrasts <0.0001). 

Results of chi-square and *t*-tests and linear regression of relationships between weight satisfaction and health behaviors, chronic conditions, and health indicators revealed that, compared to those who were satisfied with their weight, men and women who were dissatisfied with their weight were significantly more likely to report having to eat either more or less than what they would like in order to maintain their weight, dieted more, were more likely to yo-yo diet, snacked more, consumed fewer meals per day, and were less active, regardless of weight status category (Table 2). Weight satisfaction also was significantly associated with numerous health indicators and chronic disease diagnoses (Table 2). Dissatisfied men and women had higher rates of hypertension, diabetes, and hypercholesterolemia, higher percent body fat, waist circumference, total cholesterol, and lower levels of physical activity and fitness, as indicted by lower treadmill time and METs than satisfied men and women.

Results of chi-square and *t*-tests and linear regression of relationships between weight satisfaction and health behaviors, chronic conditions, and health indicators stratified by BMI group revealed that within both weight satisfaction groups (i.e., satisfied and dissatisfied) and for both genders, those who were overweight and obese consistently reported having to eat more or having to eat less than what they would like to maintain weight, more dieting and yo-yo dieting, and were less likely to be active than their normal weight counterparts. A significant relationship between weight status and number of meals per week emerged for men (both satisfied and dissatisfied) but not women, with overweight and obese men consuming fewer per week than normal weight men. Among dissatisfied women, there was a significant relationship between weight status and snacking, with dissatisfied overweight and obese women reporting snacking more than normal weight women. Finally, obese men who reported being satisfied with their weight had the highest proportion of current smokers and moderate/heavy alcohol consumers. Dissatisfied overweight and obese women had the lowest proportion of moderate to heavy drinkers (Tables 3 and 4). Within weight satisfaction groups, results for chi-square and *t*-tests demonstrated that overweight and obese men and women had consistently poorer health than those who were normal weight, with overweight and obese dissatisfied men and women having the poorest health as defined by the health indicators and chronic disease status shown in Tables 5 and 6.

Men and women who were dissatisfied with their weight had poorer health than their similar weight counterparts who were satisfied with their weight (Tables 5 and 6). For example, 29.8 overweight satisfied men were diagnosed with hypercholesterolemia compared with 36.4% of dissatisfied overweight men. Furthermore, dissatisfied overweight or obese men and women had the highest rates of hypertension, diabetes, and hypercholesterolemia and they presented with higher %body fat, waist circumference, fasting glucose, diastolic blood pressure, total cholesterol, and lower treadmill time and maximal METs. When comparing satisfied and dissatisfied men and women of similar weight status we observed significantly higher proportions reporting having to eat more or less to maintain weight, inactivity, higher dieting frequency, and yo-yo dieting among those who were dissatisfied. For example, 55.3% of dissatisfied overweight women reported a history of yo-yo dieting compared to 25% among satisfied overweight women. Weight satisfaction remained significantly positively associated with eating just enough to maintain weight, less dieting, no yo-yo dieting, eating at least 3 meals per day, being moderate to vigorously active, and never smoking among both men and women in multivariate regression analyses that adjusted for age, hypertension, myocardial infarction, stroke, cancer, diabetes, hypercholesterolemia, and all other health behaviors (Table 7).

## 5. Discussion

The epidemic of overweight and obesity observed and well-chronicled in the United States [21] is now spreading throughout the world [22–24]. At the same time these ominous trends are emerging, and we also see an upsurge in dieting and changes in meal and snacking patterns [25, 26]. Concern has been expressed about the possible consequences of the “normalization” of overweight status as a greater proportion of the population become overweight. In the ACLS, contrary to these concerns, a strong association was observed between weight dissatisfaction and weight-related health behaviors, especially dieting; the differences were larger in women than men. In addition, weight dissatisfaction was associated with adverse health indicators and chronic disease diagnoses, such as hypertension, diabetes, and hypercholesterolemia. While these findings do not provide insight into the temporality or causality of these relationships, they provide compelling evidence for a strong association between weight satisfaction and important health behaviors and outcomes. 

Adults who were dissatisfied with their weight were more likely to report eating more or less than they would like to maintain weight and higher dieting and yo-yo dieting frequency. Here the issues of satiety, dieting, and weight satisfaction intersect. Of note, ~31% of women were dieting frequently, as compared with ~18% of men. However, in general, women in this cohort had more favorable profiles of adiposity and fat distribution than men, which may explain their lower-than-population-average rate of dieting [27, 28]. However, women were more likely than men to be dissatisfied with their weight at any relative weight, even if they actually fell in the normal weight category. Gender differences in body image and ideals of thinness for women versus men likely account for this difference [1, 16]. 

There is much debate in the literature about the meal and snacking patterns that are optimal for weight control [29–31]. Studies of dietary patterns typically have focused on the positive influences of meal frequency and timing of meals [29, 30]. Smaller, more frequent meals have been shown to be positively associated with weight loss [29, 31]. Eating fewer meals per day, with most consumption occurring during evening hours, has been associated with greater intake of calories and higher BMI [29]. Reports that conflict with these findings suggest no relationship between percent of energy from evening meals and weight status [32, 33]. Current findings suggest that relationships between frequency of eating and body weight may be more complicated than is typically portrayed in the literature. Both men and women who were dissatisfied with their weight tended to snack more and consume fewer meals, but there were important gender differences. Our findings demonstrate that meal skipping is more common among overweight and obese men. There is evidence in the literature of relationships between breakfast skipping and weight gain [29]. Meal skipping and eating more or less than desired to maintain weight may signify poor regulation of energy intake and may contribute to a net excess in total caloric consumption and excessive weight gain over time. Among men, there was a more consistent relationship with meal frequency across weight status and weight satisfaction groups. Among women, the relationships were more consistent for snacking frequency but overweight and obese dissatisfied women snacked more than all other groups. It may be that dissatisfied men and women skip meals as a strategy to lose weight and that women in this cohort tended to compensate by snacking more. Further study of the role of meal and snack frequency in overall satiety and satisfaction with current weight would improve our understanding of optimal meal patterns for weight control. 

Our results indicate that men and women who are more satisfied with their weight tend to engage in more physical activity. Individuals who are fit, or become and stay fit, may be evincing motivation that is different in important ways than the motivation of those who are dieting primarily to lose weight, [34–36] though image also may matter as a motivational issue associated with efforts aimed primarily toward fitness (rather than weight control) [37]. It is also possible that these individuals may use physical activity as a means to control weight. Physical activity has been shown to increase self-esteem and body image. Perhaps people who are active, regardless of actual weight category, have a more positive outlook on their weight (i.e., they are satisfied). Active individuals may look beyond weight to focus on what their body can accomplish (e.g., participation in sports activities) and actual weight may not be as important to them. 

 Overweight and obese men were more likely to be current smokers and moderate/heavy drinkers and this trend was most striking among obese men who reported being satisfied with their weight. It is possible that these findings reflect a fatalistic approach to health or that health concerns do not influence their perceptions of themselves or their behavior. Qualitative studies on factors that influence food choice have demonstrated that health is often a less prominent influence on behavior than other values that dominate decision making processes (e.g., convenience, self-image) [38, 39]. It is possible that similar value negotiations influence response to one's weight. Further study of perceptions and health behaviors among obese men could provide insight into these findings.

Strengths of the study are its large sample size and detailed information on weight, anthropometrics, fitness, and health outcomes. Due to the cross-sectional nature of the analyses, one limitation is that temporality of the relationships between weight satisfaction and health indicators cannot be established. It is unclear whether an adverse health indicator or chronic disease diagnosis may be contributing to the feelings of dissatisfaction with weight, or whether the stress of being dissatisfied may be contributing to the adverse health status. Stress is known to be involved in the etiology of many chronic diseases, such as hypertension, diabetes, and cancer [40, 41]. Thus, examining the relationship of satisfaction and stress and the role of stress in mediating effects of weight dissatisfaction on chronic disease warrants further attention. Limitations also include the homogeneity of the mostly white, educated, and middle-to-upper-income study sample. Other populations groups such as African-American or Native-American populations have higher prevalence of obesity and different cultural attitudes toward body size [42–44]. It is possible that weight satisfaction may lead to different behaviors and outcomes in these populations. The significant findings in this lower-risk population serve to highlight the importance of examining the relationship between weight satisfaction, health behaviors, and health outcomes in populations at higher risk of obesity and related health conditions. 

This study demonstrated that greater satisfaction with one's weight is associated with healthier diet and physical activity behaviors and better health status compared to counterparts with similar BMI. It could be that those in the satisfied group who were overweight were actually in better health and trending toward normal weight. Longitudinal studies that examine these relationships would greatly improve our ability to develop effective intervention strategies to prevent development of chronic diseases. Weight satisfaction provides important insights into relationships between perceptions, behavior, and health that can be used to frame future research and intervention efforts. 

## Figures and Tables

**Table 1:** Participants' characteristics by gender, Aerobics Center Longitudinal Study, 1987–2002 (*n* = 19,003)*.

	Men (*n* = 14,408)^†,‡^	Women (*n* = 4595)
Continuous variables (mean (±SD))^§^		
Age (years)	45.7 (9.6)	44.6 (10.2)
Percent body fat (%)	21.8 (6.3)	26.7 (6.9)
Waist circumference (cm)	94.2 (10.8)	74.4 (11.2)
Treadmill time (minutes)	18.9 (5.0)	14.3 (4.6)
Maximal METs^¶^	12.1 (2.5)	10.0 (2.1)
Alcohol drinking (g/day)	13.5 (18.0)	7.5 (11.5)
Body weight (kg)	85.9 (13.7)	63.2 (11.7)
Goal weight (kg)^*⨀*^	80.1 (9.0)	57.0 (6.3)
Weight satisfaction**	5.8 (7.7)^†,‡^	6.2 (7.7)^†,‡^
Relative weight (BMI in kg/m^2^)^††^	26.7 (3.8)	23.3 (4.0)
Relative goal weight (BMI in kg/m^2^)^‡‡^	24.9 (2.1)	21.0 (1.9)
Relative weight satisfaction^§§^	1.8 (2.4)^†,‡^	2.3 (2.9)^†,‡^
Categorical variables (*n* (%))^¶¶^	Men (*n* = 14,408)	Women (*n* = 4595)
Weight status		
Underweight (<18.5 kg/m^2^)	17 (0.12)	189 (3.4)
Normal weight (18.5–24.9 kg/m^2^)	5082 (35.2)	3372 (70.5)
Overweight (25.0–29.9 kg/m^2^)	7009 (49.6)	898 (18.8)
Obese (≥30 kg/m^2^)	2317 (16.1)	325 (6.8)
Hypertension^*⨀⨀*^	4680 (32.5)	746 (16.2)
Myocardial infarction or stroke***	191 (1.3)	22 (0.5)
Cancer	695 (4.8)	318 (6.9)
Diabetes	495 (3.4)	97 (2.1)
Hypercholesterolemia^†††^	4519 (31.4)	1112 (24.2)
Physical activity level^‡‡‡^		
Inactive	4149 (28.8)	1173 (25.5)
Moderate	6814 (47.3)	2396 (52.1)
Active	3445 (23.9)	1026 (22.3)
Tobacco use^§§§^		
Never	7827 (54.3)	3013 (65.6)
Former	4626 (32.1)	1260 (27.4)
Current	1955 (13.6)	322 (7.0)
Eat more to maintain weight (yes)^¶¶¶^	1912 (13.3)	458 (10.0)
Dieting (more)^*⨀⨀⨀*^	2569 (17.8)	1457 (31.7)
Snacking		
0–6 per week	5185 (56.1)	1259 (40.6)
7+ per week	4062 (43.9)	1843 (59.4)
Meal frequency		
0–13 per week	1282 (9.0)	518 (11.1)
14–20 per week	8279 (58.3)	2271 (48.8)
21+ per week	4647 (32.7)	1865 (40.1)

*N applies for all data except *N* = 13870 for percent body fat and *N* = 13514 for waist circumference in men, and *N* = 4466 for percent body fat, *N* = 2840 for waist circumference in women; *N* does not include those in the underweight category. This data is shown for illustration but was not included in any of the analyses.

^†^Difference is significant (i.e., Ho: Satisfaction = 0) at *P* < .0001.

^‡^Difference in weight satisfaction between the genders is significant at *P* < .01.

^§^Data are means and standard deviations (SD); differences between the genders are significant at *P* < .0001 for all variables.

^¶^Maximal metabolic equivalent tasks achieved during treadmill test.

^*⨀*^Goal weight is that weight reported by the subject to be his/her ideal as of the first examination from 1987 to 2002.

**Weight Satisfaction = the Measured weight minus (−) the goal weight in kg.

^††^BMI = body mass index, a measure of relative weight is described by this formula: weight (kg)/height (m)^2^.

^‡‡^Goal weight expressed in BMI units (kg/m^2^).

^§§^Weight satisfaction expressed in BMI units (kg/m^2^).

^¶¶^All categorical variables reported as number with characteristic and percent of total (%); differences between genders significant at *P* < .0001 for all variables.

^*⨀⨀*^Defined as the history of hypertension or resting systolic/diastolic blood pressure ≥140/90 mmHg.

***Defined as any personal history of myocardial infarction or stroke; cancer, or diabetes including insulin use, or fasting blood glucose ≥7.0 mmol/L (126 mg/dL).

^†††^Defined as the history of hypercholesterolemia or fasting total cholesterol level ≥6.2 mmol/L (240 mg/dL).

^‡‡‡^Activity, from leisure-time physical activity questionnaire, inactive defined as no regular leisure-time activity; moderate defined as some participation in regular leisure-time activity, or walking, jogging, or running up to 10 miles per week; active defined as walking, jogging, or running more than 10 miles per week.

^§§§^Tobacco use defined as Never = never smoked cigarettes; Former = previously smoked cigarettes; Current = currently smoked cigarettes based on self-reported smoking habit questionnaire.

^¶¶¶^Eat more to maintain weight is coded as No = generally eating less or just what he/she wants to maintain weight; Yes = generally having to eat more than what he/she wants to maintain weight.

^*⨀⨀⨀*^Diet frequency is coded as Less = subject reports never, rarely, or only sometimes dieting; More = subject reports often or always dieting.

**Table 2:** Relationships between body weight satisfaction* and weight-related health behaviors, health indicators, and chronic disease diagnoses among women (*n* = 4595) and men (*n* = 14,408) in the Aerobics Center Longitudinal Study, 1987–2002.

Weight related behaviors^†^, chronic disease diagnoses^†^, and health indicators^‡^	WOMEN (*n* = 4595)^§^			MEN (*n* = 14,408)^¶^		
	Satisfied (*n* = 2189)	Dissatisfied (*n* = 2406)	*P* ^⨀^	Satisfied (*n* = 7121)	Dissatisfied (*n* = 7287)	*P* ^⨀^
Eating to maintain weight**						
Eat less	1138 (52.0)	1599 (66.5)		2747 (38.6)	3700 (50.8)	
Eat just	923 (42.2)	477 (19.8)	**<.0001**	3804 (53.4)	2242 (30.8)	**<.0001**
Eat more	128 (5.8)	330 (13.7)		576 (9.0)	1345 (18.5)	
Dieting frequency (more)^††^	511 (23.3)	946 (39.3)	**<.0001**	960 (13.5)	1609 (22.1)	**<.0001**
Yo-yo dieting (yes)^‡‡^	70 (17.4)	135 (43.0)	**<.0001**	131 (8.4)	282 (26.7)	**<.0001**
Snacking 7+ times per week	806 (57.2)	998 (63.2)	**.02**	1826 (41.8)	2233 (45.8)	**<.0001**
Meal frequency						
0–13 meals per week	230 (10.7)	272 (11.7)		543 (7.7)	739 (10.3)	
14–20 meals per week	1007 (46.9)	1166 (50.0)	**.02**	3851 (54.9)	4419 (61.6)	**<.0001**
≥21 meals per week	908 (42.3)	892 (38.3)		2619 (37.3)	2020 (28.1)	
Activity level^§§^						
Inactive	452 (20.6)	721 (30.0)		1566 (22.0)	2583 (35.4)	
Moderate	162 (53.1)	1234 (51.3)	**<.0001**	3418 (48.0)	3396 (46.6)	**<.0001**
Active	575 (26.3)	451 (18.7)		2137 (30.0)	1308 (17.9)	
Tobacco use^¶¶^						
Never	1484 (67.8)	1529 (63.5)		4058 (57.0)	3769 (51.7)	
Former	570 (26.0)	690 (28.7)	**.006**	2201 (31.0)	2425 (33.3)	**<.0001**
Current	135 (6.2)	187 (7.8)		862 (12.0)	1093 (15.0)	
Alcohol consumption^*⨀⨀*^						
None	703 (32.1)	934 (38.9)		1811 (25.4)	1975 (27.1)	
Light	1108 (50.6)	1087 (45.2)	**<.0001**	4399 (61.8)	4282 (58.8)	**.0009**
Moderate/Heavy	378 (17.3)	385 (16.0)		434 (6.1)	1030 (14.1)	
Chronic disease diagnoses						
Hypertension***	293 (13.4)	453 (18.8)	**<.0001**	1747 (24.5)	2933 (40.3)	**<.0001**
Myocardial infarction or stroke^†††^	7 (.3)	15 (.6)	.14	98 (1.4)	93 (1.3)	.60
Cancer^†††^	155 (7.1)	163 (6.8)	.68	376 (5.3)	319 (4.4)	**.01**
Diabetes^†††^	31 (1.4)	66 (2.7)	**.002**	146 (2.1)	349 (4.8)	**<.0001**
Hypercholesterolemia^‡‡‡^	433 (19.8)	679 (28.2)	**<.0001**	1863 (26.2)	2656 (36.5)	**<.0001**
Health Indicators^‡^						
Body mass index (kg/m^2^)	21.0 (1.6)	25.8 (4.1)	**<.0001**	24.3 (2.0)	29.1 (3.6)	**<.0001**
Percent body fat (%)	22.7 (5.4)	30.4 (5.9)	**<.0001**	18.2 (4.9)	25.2 (4.4)	**<.0001**
Waist circumference (cm)	68.7 (8.4)	79.4 (10.9)	**<.0001**	87.6 (5.8)	100.7 (7.5)	**<.0001**
Treadmill time (minutes)	15.9 (4.5)	12.9 (4.1)	**<.0001**	20.8 (4.6)	17.0 (4.0)	**<.0001**
Maximal METs^§§§^	10.7 (2.1)	9.3 (1.9)	**<.0001**	13.1 (2.4)	11.2 (1.9)	**<.0001**
Fasting glucose (mg/dL)	92.7 (13.3)	95.6 (15.4)	**<.0001**	98.4 (14.5)	102.8 (19.3)	**<.0001**
Systolic blood pressure (mmHg)	111.2 (13.9)	114.9 (14.5)	**<.0001**	120.4 (12.9)	124.1 (13.1)	**<.0001**
Diastolic blood pressure (mmHg)	74.6 (9.5)	78.0 (9.7)	**<.0001**	80.0 (9.0)	83.8 (9.6)	**<.0001**
Total cholesterol (mg/dL)	193.4 (36.7)	204.7 (39.5)	**<.0001**	202.3 (36.4)	215.3 (41.2)	**<.0001**

*Weight satisfaction is based on the difference between measured and goal weight (kg) or relative weight (kg/m^2^).

^†^Data presented as number of participants with the characteristic and the % of column total for that variable represented (%).

^‡^Data presented are means and standard deviations (SD).

^§^ *N* applies for all data except *N* = 403 for yo-yo dieting, *N* = 1408 for snacking frequency, and *N* = 2145 for meal frequency in satisfied women and *N* = 314 for yo-yo dieting, *N* = 1580 for snacking frequency, and *N* = 2330 for meal frequency in dissatisfied women and  *N* = 2136 for percent body fat and *n* = 1340 for waist circumference for satisfied women and *n* = 2330 for percent body fat and *N* = 1500 for waist circumference for dissatisfied women.

^¶^ *N* applies for all data except *N* = 1565 for yo-yo dieting, *N* = 4368 for snacking frequency, and *N* = 7013 for meal frequency for satisfied men and *N* = 1055 for yo-yo dieting, *N* = 4872 for snacking frequency, and *N* = 7178 for meal frequency in dissatisfied men and *N* = 6827 for percent body fat and *n* = 6658 for waist circumference for satisfied men and *n* = 7043 for percent body fat and *N* = 6856 for waist circumference for dissatisfied men.

^*⨀*^Significance of chi-square or Fisher's exact test for categorical and of *t*-tests for continuous variables: comparisons of behaviors, health indicators, and diagnoses between weight satisfaction columns (satisfied or dissatisfied).

**Eating to maintain weight coded as Less = generally eating less than what he wants to maintain weight; Just = eating just the amount he/she wants to maintain weight; More = generally having to eat more than what he/she wants to maintain weight.

^††^Diet frequency coded as Less = never, rarely, or only sometimes dieting; More = often or always dieting.

^‡‡^Yo-yo dieting coded as: “are you really a yo-yo dieter?” Yes or No.

^§§^Activity, from leisure-time physical activity questionnaire, inactive defined as no regular leisure-time activity; moderate defined as some participation in regular leisure-time activity, or walking, jogging, or running up to 10 miles per week; active defined as walking, jogging, or running more than 10 miles per week.

^¶¶^Tobacco use defined as Never = never smoked cigarettes; Former = previously smoked cigarettes; Current = currently smoked cigarettes based on self-reported smoking habit questionnaire.

^*⨀⨀*^alcohol consumption defined as none, light corresponds to <15 grams per day for women and <30 grams per day for men or moderate/heavy corresponds to ≥15 grams per day for women or ≥30 grams per day for men.

***Defined as the history of hypertension or resting systolic/diastolic blood pressure ≥140/90 mmHg.

^†††^Defined as any personal history of myocardial infarction or stroke; cancer, or diabetes including insulin use, or fasting blood glucose ≥7.0 mmol/L (126 mg/dL).

^‡‡‡^Defined as the history of hypercholesterolemia or fasting total cholesterol level ≥6.2 mmol/L (240 mg/dL).

^§§§^Maximal metabolic equivalent tasks achieved during treadmill test.

**Table 3:** Relationships between body weight satisfaction by overweight status and weight-related health behaviors, Aerobics Center Longitudinal Study, 1987–2002: men (*n* = 14,408)*.

Weight related behaviors^†^	Satisfied (*n* = 7121)^‡^				Dissatisfied (*n* = 7287)			
	Normal Weight^§^ (*n* = 4583)	Overweight (*n* = 2493)	Obese (*n* = 45)	*P* ^¶^	Normal Weight (*n* = 499)	Overweight (*n* = 4516)	Obese (*n* = 2272)	*P* ^¶^
Eating to maintain weight^*⨀*,a,b,c ^								
Eat less	1597 (34.9)	1132 (45.4)	18 (40.0)		245 (49.1)	2297 (50.9)	1158 (51.0)	
Eat just enough	2676 (58.4)	1107 (44.4)	24 (53.3)	**<.0001**	194 (38.9)	1476 (32.7)	572 (25.2)	**<.0001**
Eat more	310 (6.8)	254 (10.2)	3 (6.7)		60 (12.0)	743 (16.5)	542 (23.9)	
Dieting frequency (more)^∗∗,a,b ^	551 (12.0)	399 (16.0)	10 (22.2)	**<.0001**	90 (18.0)	946 (21.0)	573 (25.2)	**<.0001**
Yo-yo dieting (yes)^††,a,b^	60 (5.5)	70 (15.3)	1 (16.7)	**<.0001**	16 (15.2)	168 (23.6)	98 (41.2)	**<.0001**
Snacking 7+ times per week?^b^	1174 (43.1)	640 (39.8)	12 (35.3)	.08	144 (48.0)	1288 (43.3)	801 (50.1)	**<.0001**
Meal frequency^ b^								
0–13 meals per week	324 (7.18)	216 (8.8)	3 (6.7)		41 (8.4)	445 (10.0)	253 (11.3)	
14–20 meals per week	2401 (53.2)	1422 (57.9)	28 (62.2)	**<.0001**	279 (56.9)	2752 (61.8)	1388 (62.2)	**.003**
≥21 meals per week	1785 (39.6)	820 (33.4)	14 (31.1)		170 (34.7)	1258 (28.2)	592 (26.5)	
Activity level^‡‡,a,b,c^								
Inactive	971 (21.2)	587 (23.6)	8 (17.8)		150 (30.1)	1449 (32.1)	984 (43.3)	
Moderate	2106 (46.0)	1283 (51.5)	29 (64.4)	**<.0001**	206 (41.3)	2170 (48.1)	1020 (44.9)	**<.0001**
Active	1506 (32.9)	623 (25.0)	8 (17.8)		143 (28.7)	897 (19.9)	268 (11.8)	
Tobacco use^§§,c^								
Never	2695 (58.8)	1338 (53.7)	25 (55.6)		284 (56.9)	2359 (52.2)	1126 (49.6)	
Former	1408 (30.7)	786 (31.5)	7 (15.6)	**<.0001**	159 (31.9)	1493 (33.1)	773 (34.0)	**.0084**
Current	480 (10.5)	369 (14.8)	13 (29.0)		56 (11.2)	664 (14.7)	373 (16.4)	
Alcohol consumption^¶¶^								
None	1191 (26.0)	605 (24.3)	15 (33.3)		124 (24.9)	1157 (25.6)	694 (30.6)	
Light	2862 (62.5)	1516 (60.8)	21 (46.7)	**.0003**	314 (62.9)	2714 (60.1)	1254 (55.2)	**<.0001**
Moderate/Heavy	530 (11.6)	372 (14.9)	9 (20.0)		61 (12.2)	645 (14.3)	324 (14.3)	

*N applies to all data except *N* = 1565 yo-yo dieting, *N* = 4368 snacking frequency, and *N* = 7013 meal frequency for satisfied men and *N* = 1055 yo-yo dieting, *N* = 4872 snacking frequency, and *N* = 7178 meal frequency in dissatisfied men.

^†^Categorical variables reported as number of participants with characteristic and % of column total for variable represented (%).

^‡^Weight satisfaction based on the difference between measured and goal weight (kg) or relative weight (kg/m^2^).

^§^BMI = body mass index, a measure of relative weight is described by this formula: weight (kg)/height (m)^2^ categories; *N* = normal weight classified as BMI of 18.5–24.9; OW = overweight classified as BMI 25–29.9 kg/m^2^; Obese classified as BMI ≥ 30 kg/m^2^.

^¶^Significance of chi-square or Fisher's exact test: comparisons of weight related behaviors across BMI groups within weight satisfaction columns (satisfied or dissatisfied). (^a^Significance *P* < .05 of chi-square or Fisher's exact test: comparison of proportions for each behavior between normal weight satisfied men and normal weight dissatisfied men; significant comparisons presented in bold text. ^b^Significance at *P* < .05 of chi-square or Fisher's exact test: comparison of proportions for each behavior between overweight satisfied men and overweight dissatisfied men; significant comparisons presented in bold text. ^c^Significance at *P* < .05 of chi-square or Fisher's exact test: comparison of proportions for each behavior between obese satisfied men and obese dissatisfied men; significant comparisons are presented in bold text).

^*⨀*^Eating to maintain weight coded as Less = generally eating less than what he wants to maintain weight; Just = eating just the amount he/she wants to maintain weight; More = generally having to eat more than what he/she wants to maintain weight.

**Diet frequency coded as Less = never, rarely, or only sometimes dieting; More = often or always dieting.

^††^Yo-yo dieting coded as “are you really a yo-yo dieter?” Yes or No.

^‡‡^Activity, from self-reported leisure-time physical activity questionnaire, inactive defined as no regular leisure-time activity; moderate defined as some participation in regular leisure-time activity, or walking, jogging, or running less than 10 miles per week; active defined as walking, jogging, or running 10 miles or more per week.

^§§^Tobacco use defined as Never = never smoked cigarettes; Former = previously smoked cigarettes; Current = currently smoked cigarettes based on self-reported smoking habit questionnaire.

^¶¶^Alcohol consumption defined as none, light which corresponds to <30 grams per day or moderate/heavy which corresponds to ≥30 grams per day.

**Table 4:** Relationships between body weight satisfaction by overweight status and weight-related health behaviors, Aerobics Center Longitudinal Study, 1987–2002: women (*n* = 4595)*.

Weight related behaviors^†^	Satisfied (*n* = 2189)^‡^				Dissatisfied (*n* = 2406)			
	Normal Weight^§^ (*n* = 2147)	Overweight (*n* = 42)	Obese (*n* = 0)	*P* ^¶^	Normal Weight (*n* = 1225)	Overweight (*n* = 856)	Obese (*n* = 325)	*P* ^¶^
Eating to maintain weight^*⨀*,a,b^								
Eat less	1115 (51.9)	23 (54.8)	—		795 (64.9)	582 (68.0)	222 (68.3)	
Eat just	907 (42.2)	16 (38.1)	—	.84	306 (25.0)	133 (15.5)	38 (11.7)	**<.0001**
Eat more	125 (5.8)	3 (7.1)	—		124 (10.1)	141 (16.5)	65 (20.0)	
Dieting frequency (more)^∗∗,a^	496 (23.1)	15 (35.7)	—	.06	425 (34.7)	365 (42.6)	156 (48.0)	**<.0001**
Yo-yo dieting (yes)^††,a^	69 (17.3)	1 (25.0)	—	.69	66 (34.4)	47 (55.3)	22 (59.5)	**.0005**
Snacking 7+ times per week?	794 (57.3)	12 (52.2)	—	.62	458 (58.3)	365 (63.2)	148 (68.2)	**.02**
Meal frequency^ a^								
0–13 meals per week	226 (10.7)	4 (10.0)	—		132 (11.2)	98 (11.81)	42 (13.3)	
14–20 meals per week	987 (46.9)	20 (50.0)	—	.91	615 (51.9)	395 (47.6)	156 (49.4)	.32
≥21 meals per week	892 (42.4)	16 (40.0)	—		437 (36.9)	337 (40.6)	118 (37.3)	
Activity level^‡‡,a^								
Inactive	438 (20.4)	14 (33.3)	—		326 (26.6)	262 (30.6)	133 (40.9)	
Moderate	1137 (53.0)	25 (59.5)	—	**.008**	629 (51.4)	458 (53.5)	147 (45.2)	**<.0001**
Active	572 (26.6)	3 (7.1)	—		270 (22.0)	136 (15.9)	45 (13.9)	
Tobacco use^§§,a^								
Never	1456 (67.8)	28 (66.77)	—		768 (62.9)	543 (63.4)	218 (67.1)	
Former	560 (26.1)	10 (23.8)	—	.65	363 (29.6)	245 (28.6)	82 (25.2)	.63
Current	131 (6.1)	4 (9.5)	—		94 (7.7)	68 (7.9)	25 (7.7)	
Alcohol consumption^¶¶^								
None	688 (32.0)	15 (35.7)	—		424 (34.6)	349 (40.8)	161 (49.5)	
Light	1091 (50.8)	17 (40.5)	—	.35	586 (47.8)	369 (43.1)	132 (40.6)	**<.0001**
Moderate/Heavy	368 (17.14)	10 (23.81)	—		215 (17.6)	138 (16.1)	32 (9.9)	

*N applies for all data except *N* = 403 for yo-yo dieting, *N* = 1408 for snacking frequency, and *N* = 2145 for meal frequency in satisfied women and *N* = 314 for yo-yo dieting, *N* = 1580 for snacking frequency, and *N* = 2330 for meal frequency in dissatisfied women.

^†^Categorical variables reported as number of participants with characteristic and the % of column total for variable represented (%).

^‡^Weight satisfaction based on the difference between measured and goal weight (kg) or relative weight (kg/m^2^).

^§^BMI = body mass index, a measure of relative weight is described by this formula: weight (kg)/height (m)^2^ categories; *N* = normal weight classified as BMI of 18.5–24.9; OW = overweight classified as BMI 25–29.9 kg/m^2^; Obese classified as BMI ≥ 30 kg/m^2^.

^¶^Significance of chi-square or Fisher's exact test: comparisons of weight related behaviors across BMI groups within weight satisfaction columns (satisfied or dissatisfied). (^a^Significance *P* < .05 of chi-square or Fisher's exact test: comparison of proportions for each behavior between normal weight satisfied men and normal weight dissatisfied men; significant comparisons presented in bold text. ^b^Significance at *P* < .05 of chi-square or Fisher's exact test: comparison of proportions for each behavior between overweight satisfied men and overweight dissatisfied men; significant comparisons presented in bold text. ^c^Significance at *P* < .05 of chi-square or Fisher's exact test: comparison of proportions for each behavior between obese satisfied men and obese dissatisfied men; significant comparisons are presented in bold text).

^*⨀*^Eating to maintain weight coded as Less = generally eating less than what he wants to maintain weight; Just = eating just the amount he/she wants to maintain weight; More = generally having to eat more than what he/she wants to maintain weight.

**Diet frequency coded as Less = never, rarely, or only sometimes dieting; More = often or always dieting.

^††^Yo-yo dieting is coded as “are you really a yo-yo dieter?” Yes or No.

^‡‡^Activity, from self-reported leisure-time physical activity questionnaire, inactive defined as no regular leisure-time activity; moderate defined as some participation in regular leisure-time activity, or walking, jogging, or running less than 10 miles per week; active defined as walking, jogging, or running 10 miles or more per week.

^§§^Tobacco use defined as Never = never smoked cigarettes; Former = previously smoked cigarettes; Current = currently smoked cigarettes based on self-reported smoking habit questionnaire.

^¶¶^alcohol consumption defined as none, light which corresponds to <15 grams per day or moderate/heavy which corresponds to ≥15 grams per day.

**Table 5:** Relationships between body weight satisfaction by overweight status and health indicators and history of chronic disease, Aerobics Center Longitudinal Study, 1987–2002: men (*n* = 14408)*.

	Satisfied (*n* = 7121)^§^				Dissatisfied (*n* = 7287)			
	Normal Weight^¶^ (*n* = 4583)	Overweight (*n* = 2493)	Obese (*n* = 45)	*P* ^⨀^	Normal Weight (*n* = 499)	Overweight (*n* = 4516)	Obese (*n* = 2272)	*P* ^⨀^
Chronic disease diagnoses^†^								
Hypertension^∗∗,a,b^	972 (21.2)	749 (30.0)	26 (57.8)	**<.0001**	125 (25.1)	1584 (35.1)	1224 (53.9)	**<.0001**
Myocardial infarction or stroke^††^	55 (1.2)	43 (1.7)	0	.14	6 (1.2)	58 (1.3)	29 (1.3)	.99
Cancer^††^	244 (5.3)	130 (5.2)	2 (4.4)	.95	23 (4.6)	201 (4.5)	95 (4.2)	.85
Diabetes^††^	67 (1.5)	74 (3.0)	5 (11.1)	**<.0001**	5 (1.0)	131 (2.9)	213 (9.4)	**<.0001**
Hypercholesterolemia^‡‡,a,b^	1105 (24.2)	743 (29.8)	15 (33.3)	**<.0001**	151 (30.3)	1645 (36.4)	860 (37.9)	**.006**
Health indicators^‡^								
Percent body fat (%)^a,b,c^	16.9 (5.0)	20.6 (4.7)	23.7 (5.5)	**<.0001**	20.2 (4.5)	23.7 (4.2)	29.2 (4.7)	**<.0001**
Waist circumference (cm)^a,b,c^	84.9 (5.7)	92.4 (5.8)	99.8 (11.5)	**<.0001**	89.4 (5.0)	97.1 (6.2)	110.2 (9.9)	**<.0001**
Treadmill Time (minutes)^a,b,c^	21.6 (4.7)	19.3 (4.5)	17.6 (4.7)	**<.0001**	20.4 (4.6)	17.9 (4.1)	14.3 (3.9)	**<.0001**
Maximal METs^§§,a,b,c^	13.5 (2.5)	12.3 (2.2)	11.5 (2.3)	**<.0001**	12.8 (2.3)	11.6 (1.9)	10.0 (1.8)	**<.0001**
Fasting glucose (mg/dL)^a^	97.1 (11.8)	100.5 (18.1)	109.6 (32.4)	**<.0001**	97.7 (13.0)	100.9 (15.5)	107.7 (26.1)	**<.0001**
Systolic blood pressure (mmHg)^b^	119.7 (13.1)	121.76 (12.6)	127.6 (12.7)	**<.0001**	120.1 (13.7)	122.9 (12.8)	127.4 (13.5)	**<.0001**
Diastolic blood pressure (mmHg)^b^	79.1 (9.1)	81.3 (9.0)	86.0 (11.4)	**<.0001**	79.8 (9.5)	82.7 (9.4)	86.7 (9.9)	**<.0001**
Total cholesterol (mg/dL)^a,b,c^	199.7 (37.8)	207.2 (39.3)	201.4 (38.8)	**<.0001**	208.1 (43.2)	215.4 (41.4)	216.7 (40.3)	**<.0001**

*N applies for all data except *N* = 6827 for percent body fat and *n* = 6658 for waist circumference for satisfied men and *n* = 7043 for percent body fat and *N* = 6856 for waist circumference for dissatisfied men.

^†^Data reported as number of participants with the characteristic and the percent of column total for that variable represented (%).

^‡^Data presented are means and standard deviations (SD).

^§^Weight satisfaction is based on the difference between measured and goal weight (kg) or relative weight (kg/m^2^).

^¶^BMI = body mass index, a measure of relative weight is described by this formula: weight (kg)/height (m)^2^ categories; *N* = normal weight classified as BMI of 18.5–24.9; OW = overweight classified as BMI 25–29.9 kg/m^2^; Obese classified as BMI ≥ 30 kg/m^2^.

^*⨀*^Significance of chi-square or Fisher's exact test for categorical and of *t*-tests for continuous variables: comparisons of chronic disease diagnoses across BMI groups within weight satisfaction columns (satisfied or dissatisfied). (^a^Significance at *P* < .05 of chi-square or Fisher's exact test for categorical variables and of *t*-tests for continuous variables: comparison of health and chronic disease indicator between normal weight satisfied men and normal weight dissatisfied men; significant comparisons presented in bold text. ^b^Significance at *P* < .05 of chi-square or Fisher's exact test for categorical variables and of *t*-tests for continuous variables: comparison of health and chronic disease indicator between overweight satisfied men and overweight dissatisfied men; significant comparisons presented in bold text. ^c^Significance at *P* < .05 of chi-square or Fisher's exact test for categorical variables and of *t*-tests for continuous variables: comparison of health and chronic disease indicator between obese satisfied men and obese dissatisfied men; significant comparisons presented in bold text.)

**Defined as the history of hypertension or resting systolic/diastolic blood pressure ≥140/90 mmHg.

^††^Defined as any personal history of myocardial infarction or stroke; cancer, or diabetes including insulin use, or fasting blood glucose ≥7.0 mmol/L (126 mg/dL).

^‡‡^Defined as the history of hypercholesterolemia or fasting total cholesterol level ≥6.2 mmol/L (240 mg/dL).

^§§^Maximal metabolic equivalent tasks achieved during treadmill test.

**Table 6:** Relationships between body weight satisfaction by overweight status and health indicators and history of chronic disease, Aerobics Center Longitudinal Study, 1987–2002: women (*n* = 4595)*.

	Satisfied (*n* = 2189)^§^				Dissatisfied (*n* = 2406)			
	Normal Weight^¶^ (*n* = 2147)	Overweight (*n* = 42)	Obese (*n* = 0)	*P* ^⨀^	Normal Weight (*n* = 1225)	Overweight (*n* = 856)	Obese (*n* = 325)	*P* ^⨀^
Chronic disease diagnoses^†^								
Hypertension**	282 (13.1)	11 (26.2)	—	**.01**	157 (12.8)	181 (21.1)	115 (35.4)	**<.0001**
Myocardial infarction or stroke^††^	6 (.3)	1 (2.4)	—	**.02**	7 (.6)	5 (.6)	3 (.9)	.76
Cancer^††^	149 (6.9)	6 (14.3)	—	.07	77 (6.3)	66 (7.7)	20 (6.2)	.40
Diabetes^††^	30 (1.4)	1 (2.4)	—	.59	22 (1.8)	26 (3.0)	18 (5.5)	**.001**
Hypercholesterolemia^‡‡,a^	420 (19.6)	13 (30.1)	—	.07	274 (22.4)	282 (32.9)	123 (37.9)	**<.0001**
Health indicators^‡^								
Percent body fat (%)^a,b^	22.6 (5.3)	27.6 (5.2)	—	**<.0001**	27.2 (4.9)	32.6 (4.5)	37.1 (4.3)	**<.0001**
Waist circumference (cm)^a^	68.5 (8.1)	81.0 (11.4)	—	**<.0001**	72.6 (6.1)	81.6 (7.2)	96.2 (11.3)	**<.0001**
Treadmill time (minutes)^a,b^	16.0 (4.5)	13.2 (5.7)	—	**<.0001**	14.3 (4.0)	12.0 (3.5)	9.6 (3.3)	**<.0001**
Maximal METs^§§,a,b^	10.7 (2.1)	9.5 (2.8)	—	**.0002**	9.9 (1.9)	8.9 (1.6)	7.8 (1.5)	**<.0001**
Fasting glucose (mg/dL)	92.6 (13.4)	95.0 (10.9)	—	.24	92.9 (8.2)	97.3 (18.3)	101.0 (23.5)	**<.0001**
Systolic blood pressure (mmHg)^b^	111.1 (13.9)	116.9 (13.4)	—	**.008**	111.9 (13.9)	116.7 (14.3)	121.5 (13.9)	**<.0001**
Diastolic blood pressure (mmHg)^b^	74.6 (9.5)	76.5 (9.5)	—	.19	74.7 (9.2)	78.1 (9.5)	82.4 (9.1)	**<.0001**
Total cholesterol (mg/dL)^a^	193. 1 (36.6)	208.4 (37.0)	—	**.008**	198.6 (38.7)	210.3 (38.9)	212.9 (40.1)	**<.0001**

*N applies for all data except for *N* = 2136 for percent body fat and *n* = 1340 for waist circumference for satisfied women and *n* = 2330 for percent body fat and *N* = 1500 for waist circumference for dissatisfied women.

^†^Data reported as number of participants with the characteristic and the percent of column total for that variable represented (%).

^‡^Data presented are means and standard deviations (SD).

^§^Weight satisfaction is based on the difference between measured and goal weight (kg) or relative weight (kg/m^2^).

^¶^BMI = body mass index, a measure of relative weight is described by this formula: weight (kg)/height (m)^2^ categories; *N* = normal weight classified as BMI of 18.5–24.9; OW = overweight classified as BMI 25–29.9 kg/m^2^; Obese classified as BMI ≥ 30 kg/m^2^.

^*⨀*^Significance of chi-square or Fisher's exact test for categorical and of *t*-tests for continuous variables: comparisons of chronic disease diagnoses across BMI groups within weight satisfaction columns (satisfied or dissatisfied). (^a^Significance at *P* < .05 of chi-square or Fisher's exact test for categorical variables and of *t*-tests for continuous variables: comparison of health and chronic disease indicator between normal weight satisfied men and normal weight dissatisfied men; significant comparisons presented in bold text. ^b^Significance at *P* < .05 of chi-square or Fisher's exact test for categorical variables and of *t*-tests for continuous variables: comparison of health and chronic disease indicator between overweight satisfied men and overweight dissatisfied men; significant comparisons presented in bold text. ^c^Significance at *P* < .05 of chi-square or Fisher's exact test for categorical variables and of *t*-tests for continuous variables: comparison of health and chronic disease indicator between obese satisfied men and obese dissatisfied men; significant comparisons presented in bold text.)

**Defined as the history of hypertension or resting systolic/diastolic blood pressure ≥140/90 mmHg.

^††^Defined as any personal history of myocardial infarction or stroke; cancer, or diabetes including insulin use, or fasting blood glucose ≥7.0 mmol/L (126 mg/dL).

^‡‡^Defined as the history of hypercholesterolemia or fasting total cholesterol level ≥6.2 mmol/L (240 mg/dL).

^§§^Maximal metabolic equivalent tasks achieved during treadmill test.

**Table 7:** Association of weight satisfaction with weight-related health behaviors among women (*n* = 4595) and men (*n* = 14,408) in the Aerobics Center Longitudinal Study, 1987–2002.

Health behavior	Men (*n* = 14,408)		Women (*n* = 4595)	
	Model 1*	Model 2^†^	Model 1*	Model 2^†^
	OR (95% CI)	OR (95% CI)	OR (95% CI)	OR (95% CI)
Eat just enough for weight maintenance	**2.49 (2.31–2.69)**	**1.66 (1.51–1.83)**	**2.66 (2.30–3.08)**	**1.71 (1.41–2.06)**
Less dieting frequency	**1.34 (1.21–1.48)**	**1.17 (1.04–1.31)**	**1.51 (1.31–1.74)**	**1.21 (1.02–1.44)**
No yo-yo dieting	**2.03 (1.84–2.25)**	**1.17 (1.03–1.32)**	**2.31 (1.89–2.83)**	**1.37 (1.06–1.77)**
Snacking <7 times per week	1.00 (0.93–1.08)	1.06 (0.97–1.16)	1.09 (0.95–1.26)	1.04 (0.88–1.24)
Meal frequency ≥21	**1.42 (1.32–1.54)**	**1.25 (1.14–1.38)**	**1.22 (1.07–1.38)**	**1.17 (1.00–1.37)**
Moderate or vigorous physical activity	**1.94 (1.79–2.10)**	**1.48 (1.35–1.63)**	**1.77 (1.53–2.04)**	**1.34 (1.13–1.60)**
Never smoker	**1.15 (1.07–1.24)**	1.07 (0.98–1.17)	**1.15 (1.01–1.31)**	**1.20 (1.03–1.40)**
Light alcohol consumption	1.06 (0.99–1.14)	0.97 (0.88–1.06)	**1.20 (1.06–1.36)**	1.06 (0.91–1.23)

The referent category for each analysis includes men and women who are dissatisfied with their body weight.

*Adjusted for age, hypertension, myocardial infarction, stroke, cancer, diabetes, hypercholesterolemia, and all other health behaviors in the table.

^†^Adjusted as for model 1 plus body mass index (kg/m^2^).
